# The first finding of (TTAGG)_n_ telomeric repeat in chromosomes of true bugs (Heteroptera, Belostomatidae)

**DOI:** 10.3897/CompCytogen.v6i4.4058

**Published:** 2012-10-05

**Authors:** Valentina G. Kuznetsova, Snejana M. Grozeva, Boris A. Anokhin

**Affiliations:** 1 Zoological Institute, Russian Academy of Sciences, Universitetskaya nab. 1, St. Petersburg 199034, Russia; 2 Institute of Biodiversity and Ecosystem Research, Bulgarian Academy of Sciences, Blvd Tsar Osvoboditel 1, Sofia 1000, Bulgaria

**Keywords:** Chromosomes, FISH, (TTAGG)_n_ telomeric repeat, true bugs, Nepomorpha, Belostomatidae, *Lethocerus patruelis*

## Abstract

Using the fluorescence *in situ* hybridization (FISH), the presence of (TTAGG)_n_ telomeric sequence was detected in the chromosomes of *Lethocerus patruelis* (Stål, 1854) belonging to the family Belostomatidae (Heteroptera: Nepomorpha). This sequence was exclusively present at the ends of chromosomes in this species. This is the first evidence of the insect-type TTAGG telomeric repeats in Heteroptera.

## Introduction

Telomeres are specific nucleoprotein structures at the ends of chromosomes and are responsible for their stability. Information on the telomere structure and function is presently available for many animals, plants and fungi (Fuchs et al. 1995, McKnight and Shippen 2004, Traut et al. 2007, Zakian 2012). The telomeres of insect species are predominantly composed of a pentanucleotide sequence repeat (TTAGG)_n_ (reviewed in Frydrychová et al. 2004). On the other hand, there are some higher taxa known to have lost this telomeric motif during their evolution, and Heteroptera are repeatedly referred to as one of such groups (Sahara et al. 1999, Frydrychová et al. 2004, Vitková et al. 2005, Lukhtanov and Kuznetsova 2010, Grozeva et al. 2011, Kuznetsova et al. 2011).

In this paper we report the molecular structure of telomeres at the physical ends of chromosomesin *Lethocerus patruelis* (Stål, 1854) (Nepomorpha: Belostomatidae).

## Material and methods

Spread chromosome preparations were made from testes of *Lethocerus patruelis* and stained using a Shiff-Giemsa method as described in Grozeva et al. (in press). The molecular structure of telomeres was investigated by fluorescence *in situ* hybridization of chromosomes (FISH) with a (TTAGG)_n_ probe. In addition, we used an 18S rDNA probe to reveal the location of ribosomal clusters, NORs, on *Lethocerus patruelis* chromosomes. In these experiments we followed the protocol described in Grozeva et al. (2011). Fluorescence images were taken with a Leica DFC 345 FX camera using Leica Application Suite 3.7 software with an Image Overlay module.

## Results

**Figures 1.**
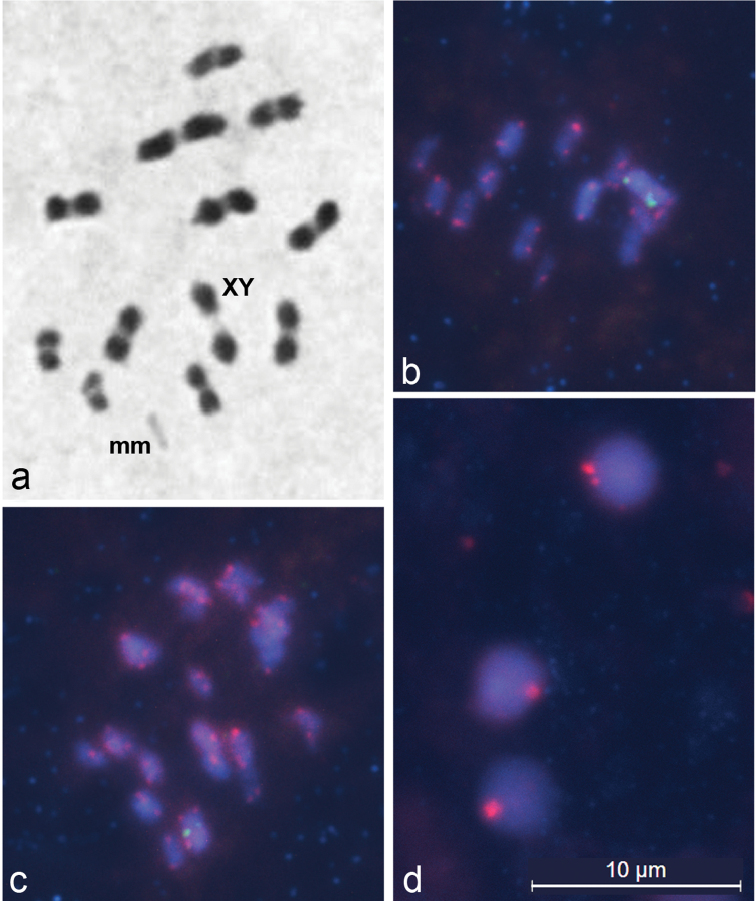
Meiotic chromosomes of *Lethocerus patruelis* subjected to standard staining (**a**) and FISH (**b–d**). **a** metaphase I showing n = 11AA + mm + XY; **b–d** representative FISH images of metaphase I chromosomes (**b, c**) and spermatids (**d**) hybridized with probes against 18S rDNA and telomeres, showing ribosomal clusters (green) on X and Y chromosomes (**b, c**), and TTAGG repeats (red) located at the ends of chromosomes (**b, c**) and clustered at the periphery of spermatid nuclei (**d**).

At first metaphases in *Lethocerus patruelis* males, 11 autosomal bivalents, each with one (sometimes two) terminal or subterminal chiasmata, a bivalent of m-chromosomes (micro-chromosomes) and a XY- pseudo-bivalent could be seen (Fig. 1a). Figures 1b-d show the results of fluorescence *in situ* hybridization with pentanucleotide (TTAGG)_n_ and 18S rDNA probes to several meiotic spreads. At metaphase nuclei, TTAGG fluorescent signals (red) are clearly seen at all chromosomal ends, whereas rDNA clusters (green) are clearly evident on the X and Y chromosomes (Fig. 1b, c). Prominent telomere clustering at the periphery of spermatid nuclei (Fig. 1d) creates one large while sometimes a small number of TTAGG signals (red).

## Discussion

The standard karyotype of *Lethocerus patruelis* males is 2n = 22A + 2m + XY as it was recently shown by Grozeva et al. (in press). We found that *Lethocerus patruelis* displayed FISH rDNA sites both on X and Y chromosomes. This is as expected since CMA_3_-staining performed by Grozeva et al. (in press) has revealed GC rich clusters (typically pointed to NORs) on the sex chromosomes in this species. In other Belostomatidae species studied in this respect, NORs are known to be located either on sex chromosomes or on a pair of autosomes, the co-generic species sometimes differing in this pattern (reviewed in Grozeva et al. 2011).

DNA of the telomeres consists of short nucleotide motifs (combinations) repeated thousands and millions of times. Comparative analysis of these motifs in various groups of organisms has shown that they are evolutionarily stable, and, having once appeared during the evolution, mark taxa and phylogenetic lineages of high rank (Traut et al. 2007).

Quite recently, Frydrychová et al. (2004) assembled and analyzed the data available on the telomeric sequences in Insecta, and, together with some original observations, they interpreted these character data in a phylogenetic framework. Conclusions in that work are largely congruent with those previously proposed by Sahara et al. (1999). The great majority of insect species share the telomeres composed of (TTAGG)_n_ repeat. Since the same telomere composition is characteristic of the rest of arthropods, the (TTAGG)_n_ telomeric motif is considered an ancestral one in Insecta. Many higher-level insect groups preserved this motif; however several orders, e.g. Dermaptera, Heteroptera, Diptera and some others, are suggested to have lost this telomeric sequence during the evolution (Sahara et al. 1999, Frydrychová et al. 2004, Vitková et al. 2005, Lukhtanov and Kuznetsova 2010).

We emphasize, however, that the problem of telomere composition in different insect orders is still not adequately explored and in most cases, the available data concern one or more species only (see Fig. 6 in Frydrychová et al. 2004). On the other hand, in one of the better studied orders, Coleoptera (data are available for more than 20 species), both (TTAGG)_n_-positive and (TTAGG)_n_-negative species have been reported (Frydrychová and Marec 2002, Frydrychová et al. 2004).

In Heteroptera, the absence of the (TTAGG)_n_ telomeric motif was firstly shown for *Halyomorpha halys* (Stål, 1855) (Pentatomidae) studied using Southern hybridization (Okazaki et al. 1993: as *Halyomorpha mista* (Uhler, 1860)) and *Pyrrhocoris apterus* (Linnaeus, 1758) (Pyrrhocoridae) subjected to both Southern hybridization and FISH (Sahara et al. 1999). On the other hand, this sequence was revealed in telomeres of non-heteropteran Hemiptera and some other Paraneoptera (Frydrychova et al., 2004).

Originally proposed by Sahara et al. (1999) and accepted at a later time by other authors (Frydrychova et al. 2004, Vitková et al. 2005, Lukhtanov and Kuznetsova 2010), the hypothesis for the loss of (TTAGG)_n_ sequence in true bugs has received further support owing to the discovery of Grozeva et al. (2011) that five more species studied by FISH and Dot-blot hybridization are also (TTAGG)_n_- negative. Based on evidence provided by Okazaki et al. (1993), Sahara et al. (1999) and Grozeva et al. (2011), (TTAGG)_n_ motif is known to be absent in seven species of true bugs. These species represent phylogenetically distant families, such as Pentatomidae (*Halyomorpha halys*, *Eurydema oleracea* (Linnaeus, 1758), *Graphosoma lineatum* (Linnaeus, 1758)) and Pyrrhocoridae (*Pyrrhocoris apterus*) belonging to the infraorder Pentatomomorpha and also Miridae (*Deraeocoris rutilus* (Herrich-Schaffer, 1838), *Megaloceroea recticornis* (Geoffroy, 1785)) and Cimicidae (*Cimex lectularius* (Linnaeus, 1758) belonging to the infraorder Cimicomorpha.

Our results of FISH with a (TTAGG)_n_ probe strongly demonstrated that (TTAGG)_n_ sequence was located at the telomeres of all chromosomes in *Lethocerus patruelis*. The finding of the insect-type (TTAGG)_n_ telomeric motif in *Lethocerus patruelis* is thus clearly indicative of the heterogeneity of Heteroptera in telomere organization. The family Belostomatidae, to which this species belongs, is classified within the infraorder Nepomorpha (or true water bugs). The data on telomeres imply that true water bugs preserved the plesiomorphic telomere structure, whereas Cimicomorpha and Pentatomomorpha have the apomorphic state of this character, which can be considered a synapomorphy of these infraorders. This conclusion is consistent with the generally accepted opinion that Cimicomorpha and Pentatomomorpha represent a monophyletic lineage, and Nepomorpha has a basal position within Heteroptera (Wheeler et al. 1993; Mahner 1993, Scherbakov and Popov 2002, Xie et al. 2008, Weirauch and Schuh 2011).
